# Microchimerism, PERV and Xenotransplantation

**DOI:** 10.3390/v15010190

**Published:** 2023-01-10

**Authors:** Joachim Denner

**Affiliations:** Institute of Virology, Free University Berlin, 14163 Berlin, Germany; joachim.denner@fu-berlin.de

**Keywords:** xenotransplanation, microchimerism, porcine endogenous retroviruses, detection methods

## Abstract

Microchimerism is the presence of cells in an individual that have originated from a genetically distinct individual. The most common form of microchimerism is fetomaternal microchimerism, i.e., cells from a fetus pass through the placenta and establish cell lineages within the mother. Microchimerism was also described after the transplantation of human organs in human recipients. Consequently, microchimerism may also be expected in xenotransplantation using pig cells or organs. Indeed, microchimerism was described in patients after xenotransplantations as well as in non-human primates after the transplantation of pig organs. Here, for the first time, a comprehensive review of microchimerism in xenotransplantation is given. Since pig cells contain porcine endogenous retroviruses (PERVs) in their genome, the detection of proviral DNA in transplant recipients may be misinterpreted as an infection of the recipient with PERV. To prevent this, methods discriminating between infection and microchimerism are described. This knowledge will be important for the interpretation of screening results in forthcoming human xenotransplantations.

## 1. Introduction

The recent transplantation of a pig heart into a patient in Baltimore provided evidence for the transmission of a pig virus to a human recipient. The virus shown to be transmitted to the patient (however, there is no evidence that the virus infected human cells) was the porcine cytomegalovirus (PCMV) [1]. The name is misleading; it is actually a porcine roseolovirus, and in order to underline this, the abbreviation PCMV/PRV is used. The official International Committee on Taxonomy of Viruses (ITCV) name is suid betaherpesvirus 2 (SuBHV2) [2]. The virus is, therefore, more closely related to the human herpesviruses HHV-6 and HHV-7, not to the human cytomegalovirus (HCMV, HHV-5) [3]. 

No detailed data are as of yet available concerning the transmission of porcine endogenous retroviruses (PERVs), which are present in the genome of all pigs, to the patient. The authors mention that they did not find a transmission of PERV; however, they did not describe the method and the sensitivity of the method used [1]. Since in nearly all allotransplantation and xenotransplantation transplanting organs, microchimerism was detected (see below), this may also be expected in the Baltimore patient. Possibly screening for PERV was only performed in cell-free sera, not in the cells. Alternatively, the detection method was not sensitive enough. 

Screening for PERV in cells and tissues automatically also screens for microchimerism. PERV is approximately integrated into the genome of a pig cell 60 times [4], and therefore, screening for PERV should be 60 times more sensitive in detecting pig cells compared with screening using cellular genes [5]. However, in the case of a positive result, methods discriminating between infection of human cells and microchimerism should be used (see below).

There is agreement within the field that, at present, all possible experimental approaches to evaluate the risks posed by PERVs are already being exploited and that there is no way to definitively and reliably assess the risks posed by PERV with additional experiments [6]. Only a follow up with actual xenotransplant recipients will provide the answer. Therefore, the first clinical xenotransplantations are of great interest. 

## 2. Fetomaternal Microchimerism

The most common form of microchimerism is fetomaternal microchimerism, i.e., cells from a fetus pass through the placenta and establish cell lineages within the mother. Fetal cells have been documented to persist and multiply in the mother for several decades [7,8]. Additionally, it was shown that HLA-disparate maternal cells can persist in immunocompetent offspring well into adult life [9]. Notably, fetal micromeric cells were reported to show progenitor cell and stem cell phenotypes (for a review, see [10]).

## 3. Microchimerism after Allotransplantation

Transplantation of human organs is also associated with microchimerism, i.e., the detection of donor cells in different organs of the recipient [11,12,13,14]. On one hand, these cells are thought to be the reason for autoimmune diseases [15,16]; on the other hand, it is thought that they may help to induce donor-specific hyporesponses, leading to a survival of the transplant with reduced pharmaceutical immunosuppression or even immunological tolerance [17]. 

## 4. Microchimerism after Clinical Trials of Xenotransplantation 

Therefore, microchimerism may also be expected in xenotransplantation using the cells, tissues or organs of pigs. Indeed, microchimerism, i.e., the occurrence of pig cells, was observed in recipients of pig xenotransplants. For example, samples were taken from 23 Russian patients who had an extracorporeal splenic perfusion through spleens from healthy slaughterhouse pigs as immunotherapy for various indications [18]. The ex vivo perfusion of a pig organ (spleen, liver) also counts as xenotransplantation. The patients had 43.7 person-years (2 and 102 months) of cumulative exposure to pig cells. The long-term persistence of microchimerism in these patients was an unexpected finding, as none of the patients have been immunosuppressed since the procedure. The quantitative results obtained from an analysis of the patient samples suggested that the level of microchimerism was extremely low (less than one pig cell per 500,000 PBMCs in the majority of cases) [18]. Calculating the number of PERV copies and the number of cellular genes, the authors were able to show that the presence of PERV sequences in these patients was due to microchimerism, not due to infection [18]. 

In another clinical trial transplanting encapsulated pig islet cells into diabetic patients, no PERV transmission and no microchimerism were observed [15]. This was not surprising since the islet cells were encapsulated and their number was low in comparison with the number of cells of a whole organ. At different time points (up to 113 weeks), white blood cells (WBC) have been tested for PERV DNA, as well as WBC and plasma for PERV RNA by real-time RT-PCR. All tests were negative. In addition, using primers detecting the pig mitochondrial cytochrome oxidase (COX) gene, patients were screened for microchimerism using a cellular gene, which was also negative [19]. However, the absence of PERV DNA is proof in itself of the absence of pig cells. Since in pig cells, up to 60 PERV copies are integrated, the detection method for PERV sequences is usually up to 60 times more efficient in the detection of pig cells compared with PCR using cellular pig genes, as explained above [5]. In a second trial transplanting encapsulated pig islet cells into human patients, again, no microchimerism was observed [20], despite the fact that in a previous study analyzing the same patients using a multiplex high-resolution melting assay, microchimerism had been reported [21], indicating that porcine cells were transiently detectable in the blood stream of several transplant recipients at various time points post-transplantation. The detection of porcine sequences in the absence of PERV in this study was possible due to a multiplex high-resolution melting assay being used, detecting pig COX. Obviously this assay had superior analytical sensitivity for COX over that of PERV [18].

## 5. Microchimerism after Preclinical Trials of Xenotransplantation 

In preclinical xenotransplantation trials, the aspect of microchimerism was also studied. In one trial, transplanting pig kidneys into rhesus macaques, PERV sequences were detected in the bladder of the animals [22]. Insertion experiments confirmed that PERVs originated from porcine donor cells rather than an integrated provirus in the monkey chromosome. The presence of pig cells in the monkey bladder after renal xenotransplantation was also demonstrated using specific-porcine mitochondrial DNA gene PCR [22]. By contrast, in monkey organs after cardiac xenotransplantation, PERV genes were not detected, indicating the absence of pig cells in the monkey, i.e., absence of microchimerism [22]. 

In a preclinical trial transplanting orthotopically pig hearts into baboons, cells expressing antigens of the porcine cytomegalovirus/porcine roseolovirus (PCMV/PRV) were detected in different organs of the baboon recipient by immunohistochemistry [23]. Since up until now, there has been no evidence that PCMV/PRV can infect human and non-human primate cells, it was assumed that these cells represent disseminated pig cells, e.g., microchimerism. Using a PCR detecting PERVs, positive signals were detected in the blood of eight baboons that received orthotopic heart transplants and that had survival times between 10 and 195 days [24]. All animals were negative for antibodies against PERV, indicating that they were not infected [24]. 

PERV DNA sequences and porcine mitochondrial DNA were detected in the peripheral blood mononuclear cells of 6 out of 14 (43%) animals with heterotopic pig heart and pig kidney transplants, indicating that the detection of PERV sequences was attributable to microchimerism [25]. These conclusions were also drawn in another study [26].

## 6. Conclusions and Implications for PERV Testing

To summarize, screening for PERV infections in human recipients will be difficult due to the expected microchimerism. Therefore, this analysis has to be performed responsibly. Insertion experiments should demonstrate that human cells are infected. As described, using the long-terminal repeat (LTR) region, the integration of PERV into the host genome has to be analyzed via inverse PCR using specific primers [22]. The inverse PCR-amplified genes have to be cloned, and insert sequences should be analyzed. Next-generation sequencing (NGS) of the neighbor genes of integrated PERV proviruses can also be used to discriminate between PERV integrated in pig cells or human cells. Only sensitive methods have to be used in order to obtain reliable results.

## Data Availability

Not applicable.

## References

[1] Griffith B.P., Goerlich C.E., Singh A.K., Rothblatt M., Lau C.L., Shah A., Lorber M., Grazioli A., Saharia K.K., Hong S.N. (2022). Genetically Modified Porcine-to-Human Cardiac Xenotransplantation. N. Engl. J. Med..

[2] https://ictv.global/report/chapter/herpesviridae/herpesviridae/roseolovirus.

[3] Denner J., Bigley T.M., Phan T.L., Zimmermann C., Zhou X., Kaufer B.B. (2019). Comparative Analysis of Roseoloviruses in Humans, Pigs, Mice, and Other Species. Viruses.

[4] Fiebig U., Fischer K., Bähr A., Runge C., Schnieke A., Wolf E., Denner J. (2018). Porcine endogenous retroviruses: Quantification of the copy number in cell lines, pig breeds, and organs. Xenotransplantation.

[5] Denner J. (2021). Detection of cell-free pig DNA using integrated PERV sequences to monitor xenotransplant tissue damage and rejection. Xenotransplantation.

[6] Denner J. (2018). Why was PERV not transmitted during preclinical and clinical xenotransplantation trials and after inoculation of animals?. Retrovirology.

[7] Bianchi D.W., Zickwolf G.K., Weil G.J., Sylvester S., DeMaria M.A. (1996). Male fetal progenitor cells persist in maternal blood for as long as 27 years postpartum. Proc. Natl. Acad. Sci. USA.

[8] Evans P.C., Lambert N., Malone S., Furst D.E., Moore J.M., Nelson J.L. (1999). Long-Term Fetal Microchimerism in Peripheral Blood Mononuclear Cell Subsets in Healthy Women and Women with Scleroderma. Blood.

[9] Maloney S., Smith A., Furst D.E., Myerson D., Rupert K., Evans P.C., Nelson J.L. (1999). Microchimerism of maternal origin persists into adult life. J. Clin. Investig..

[10] Ståhlberg A., El-Heliebi A., Sedlmayr P., Kroneis T. (2018). Unravelling the biological secrets of microchimerism by single-cell analysis. Brief. Funct. Genom..

[11] Muramatsu K., Valenzuela R.G., Bishop A.T. (2003). Detection of chimerism following vascularized bone allotransplantation by polymerase chain reaction using a Y-chromosome specific primer. J. Orthop. Res..

[12] Rao A.S., Thomson A.W., Shapiro R., Starzl T.E. (1994). Chimerism after whole organ transplantation: Its relationship to graft rejection and tolerance induction. Curr. Opin. Nephrol. Hypertens..

[13] Starzl T.E., Demetris A.J., Trucco M., Zeevi A., Ramos H., Terasaki P., Rudert W.A., Kocova M., Ricordi C., Ildstad S. (1993). Chimerism and donor-specific nonreactivity 27 to 29 years after kidney allotransplantation. Transplantation.

[14] Starzl T.E., Demetris A.J., Trucco M., Murase N., Ricordi C., Ildstad S., Ramos H., Todo S., Tzakis A., Fung J.J. (1993). Cell migration and chimerism after whole-organ transplantation: The basis of graft acceptance. Hepatology.

[15] Gleicher N., Barad D.H. (2007). Gender as risk factor for autoimmune diseases. J. Autoimmun..

[16] Gilliam A.C. (2006). Microchimerism and skin disease: True-true unrelated?. J. Investig. Dermatol..

[17] Mazariegos G.V., Reyes J., Marino I.R., Demetris A.J., Flynn B., Irish W., McMichael J., Fung J.J., Starzl T.E. (1997). Weaning of immunosuppression in liver transplant recipients. Transplantation.

[18] Paradis K., Langford G., Long Z., Heneine W., Sandstrom P., Switzer W.M., Chapman L.E., Lockey C., Onions D., Otto E. (1999). Search for cross-species transmission of porcine endogenous retrovirus in patients treated with living pig tissue. The XEN 111 Study Group. Science.

[19] Morozov V.A., Wynyard S., Matsumoto S., Abalovich A., Denner J., Elliott R. (2017). No PERV transmission during a clinical trial of pig islet cell transplantation. Virus Res..

[20] Wynyard S., Nathu D., Garkavenko O., Denner J., Elliott R. (2014). Microbiological safety of the first clinical pig islet xenotransplantation trial in New Zealand. Xenotransplantation.

[21] Wynyard S., Garkavenko O., Elliot R. (2011). Multiplex high resolution melting assay for estimation of Porcine Endogenous Retrovirus (PERV) relative gene dosage in pigs and detection of PERV infection in xenograft recipients. J. Virol. Methods.

[22] Heo Y., Cho Y., Oh K.B., Park K.H., Cho H., Choi H., Kim M., Yun I.J., Lee H.J., Kim Y.B. (2019). Detection of Pig Cells Harboring Porcine Endogenous Retroviruses in Non-Human Primate Bladder After Renal Xenotransplantation. Viruses.

[23] Fiebig U., Abicht J.M., Mayr T., Längin M., Bähr A., Guethoff S., Falkenau A., Wolf E., Reichart B., Shibahara T. (2018). Distribution of Porcine Cytomegalovirus in Infected Donor Pigs and in Baboon Recipients of Pig Heart Transplantation. Viruses.

[24] Denner J., Längin M., Reichart B., Krüger L., Fiebig U., Mokelke M., Radan J., Mayr T., Milusev A., Luther F. (2020). Impact of porcine cytomegalovirus on long-term orthotopic cardiac xenotransplant survival. Sci. Rep..

[25] Moscoso I., Hermida-Prieto M., Mañez R., Lopez-Pelaez E., Centeno A., Diaz T.M., Domenech N. (2005). Lack of cross-species transmission of porcine endogenous retrovirus in pig-to-baboon xenotransplantation with sustained depletion of anti-alpha gal antibodies. Transplantation.

[26] Issa N.C., Wilkinson R.A., Griesemer A., Cooper D.K.C., Yamada K., Sachs D.H., Fishman J.A. (2008). Absence of Replication of Porcine Endogenous Retrovirus and Porcine Lymphotropic Herpesvirus Type 1 with Prolonged Pig Cell Microchimerism after Pig-to-Baboon Xenotransplantation. J. Virol..

